# Rapid fabrication of custom patient biopsy guides

**DOI:** 10.1120/jacmp.v10i4.2897

**Published:** 2009-09-02

**Authors:** Didier A. Rajon, Frank J. Bova, Yueh‐Yun Chi, William A. Friedman

**Affiliations:** ^1^ Department of Neurosurgery University of Florida University of Florida Gainesville FL USA; ^2^ Department of Epidemiology and Health Policy Research University of Florida Gainesville FL USA

**Keywords:** subtractive rapid prototyping, image‐guided surgery, stereotactic frame, biopsy guide

## Abstract

Image‐guided surgery is currently performed using frame‐based as well as frameless approaches. In order to reduce the invasive nature of stereotactic guidance and the cost in both equipment and time required within the operating room, we investigated the use of rapid prototyping (RP) technology. In our approach, we fabricated custom patient‐specific face masks and guides that can be applied to the patient during stereotactic surgery. While the use of RP machines has previously been shown to be satisfactory from an accuracy standpoint, one of our design criteria – completing the entire build and introduction into the sterile field in less than two hours – was unobtainable.^(1)^ Our primary problems were the fabrication time and the nonresistance of the built material to high‐temperature sterilization. In the current study, we have investigated the use of subtractive rapid prototyping (SRP) machines to perform the same quality of surgical guidance, while improving the fabrication time and allowing for choosing materials suitable for sterilization. Because SRP technology does not offer the same flexibility as RP in terms of prototype shape and complexity, our software program was adapted to provide new guide designs suitable for SRP fabrication. The biopsy guide was subdivided for a more efficient build with the parts being uniquely assembled to form the final guide. The accuracy of the assembly was then assessed using a modified Brown‐Roberts‐Wells phantom base by which the position of a biopsy needle introduced into the guide can be measured and compared with the actual planned target. These tests showed that: 1) SRP machines provide an average technical accuracy of 0.77 mm with a standard deviation of the mean of 0.07 mm, and 2) SRP allows for fabrication and sterilization within three‐and‐a‐half hours after diagnostic image acquisition. We are confident that technology is capable of reducing this time to less than one hour. Further tests are being conducted to determine the registration accuracy of the face mask on the patient's head under IRB‐approved trials. The accuracy of this new guidance technology will be verified by judging it against current frame‐based or frameless systems.

PACS number: 87.57.Gg

## I. INTRODUCTION

The advent of X‐ray computed tomography (CT) and magnetic resonance (MR) imaging has provided significant assistance in the pre‐surgical planning process. To aid the surgeon in his or her ability to appreciate the location of target tissues as well as their relationship to normal tissue, graphical workstations have been employed. The latest generations of workstations combined the ability to: 1) reconstruct a three‐dimensional (3D) volume dataset from a series of diagnostic images, 2) evaluate alternate surgical approaches through the manipulation of the patient‐specific 3D model, and 3) apply a virtual surgical plan to the real world patient at the time of surgery. This process is termed image‐guided surgery (IGS).^(2)^ These three fundamental steps have improved significantly over the last decades due to the increase of computational power and to a continuous improvement of engineering and technology.

State‐of‐the‐art IGS procedures can be divided into two major technologies. The first, and older, technology involves the use of a rigid frame. This technology remains popular with primary applications involving intracranial radiosurgery and biopsy. The frame‐based procedure involves the application of a stereotactic frame to the patient prior to image data acquisition.^(^ ^3^ ^,^ ^4^ ^)^ The frame remains on the patient through the entire procedure and serves to attach various apparatuses that are needed during both image acquisition and surgical procedure.^(^ ^5^ ^,^ ^6^ ^)^ Frame‐based procedures continue to provide the highest degree of accuracy and precision for IGS, but they require the patient to wear the rigid frame during the entire stereotactic procedure.

The second, and newer, technology involves a frameless approach in which fiducial landmarks are derived from the patient's own anatomical features^(7)^ or applied to the patient's skin prior to diagnostic image acquisition.^(^ ^8^ ^,^ ^9^ ^)^ The fiducials are used to coordinate the computer‐generated virtual patient model to the real‐world patient.^(^ ^10^ ^–^ ^14^ ^)^ Frameless technology has been applied to stereotactic surgery as well as stereotactic radiosurgery.^(^ ^15^ ^–^ ^17^ ^)^ Since the application of fiducial landmarks is less invasive than a fixed frame, frameless procedures allow a more relaxed time sequence and better comfort for the patient. However, it requires cumbersome and expensive instrument‐tracking equipment to be present in the operating room (OR).^(11)^ Recently, 3D ultrasound has been applied to stereotactic guidance for extracranial targeting.^(^ ^18^ ^–^ ^20^ ^)^ Time and cost limit image guidance to a small fraction of the procedures which could be made more accurate – for example, shunts, all craniotomies, and microvascular decompressions.

To reduce both discomfort and cost, we investigated the use of rapid prototyping (RP) technology to fabricate patient custom‐positioning fixtures.^(1)^ In this investigation, the image dataset provided by the diagnostic modality was used to design a face mask that conforms to the patient's anatomy allowing a unique positioning and referencing for guidance of the surgical tools. Guides were then designed and added to the mask and a three‐dimensional printer (3DP) was used to fabricate the whole fixture. It was shown that the accuracy of the technology was compatible with the use of available frameless optically guided procedures.^(1)^ However, the overall fabrication time could never be reduced to satisfy the need for clinical applications. Five to six hours was the minimum that could possibly be achieved. Furthermore, the material used in the 3DP cannot be sterilized at high temperatures (134 °C) and requires the use of low‐temperature sterilization, which typically adds an extra ten hours before the frame can be used in the OR. Other RP technologies use materials that can withstand high temperatures^(^ ^21^ ^–^ ^24^ ^)^ but they are more costly and also necessitate a longer fabrication time.

**Figure 1:**
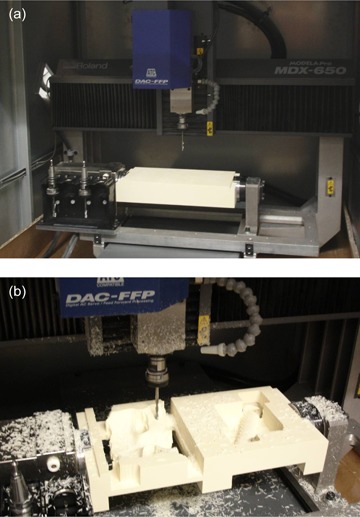
The MDX‐650 milling machine: general view (a) with a block of material ready to be tooled, and (b) as the face‐mask and the custom arm of the biopsy guide are being built out of the block.

To overcome the time and sterilization issues we investigated the use of programmable milling machines – also known as subtractive rapid prototyping (SRP) machines – to fabricate the positioning face mask as well as the different guides attached to it. These machines allow for using any tooling materials, ranging from soft composite plastics to aluminum and stainless steel. They also provide faster fabrication time and are more precise. Whereas RP technology can fabricate any object defined by a closed surface, SRP has three major limitations. First, because the object is built by removing material from an initial block, only surfaces that can be positioned so that they are facing the milling tool can be cut – no under‐cuts or hollows are allowed. Second, because the initial block of material is rectangular, complex elongated objects require a large block that would necessitate removing a lot of material and, as a consequence, a longer cutting time. Third, SRP machines are classified as 3–, 4‐, 5‐, or 6‐axis depending on how many degrees of freedom are provided by their design. With three axes, the cutting tool can only move along the three main axes; no rotation is allowed. A 4‐axis machine allows the block of material to rotate around one axis. With five or six axes, the tool can rotate around the block and can cut more complex objects. Five‐ and six‐axis machines are expensive and are not competitive with 3DP, in terms of cost. We elected to utilize the most cost‐effective technology in an effort to see if the lower‐end technology could fabricate the required mask and guide features. If we could establish the lower‐end units as being capable of the required fabrication, then it logically follows that the time required for fabrication could be reduced by more robust, more capable and more expensive models. Therefore, we decided to acquire a 4‐axis machine manufactured by Roland DG Corp. (Knoxville, TN), model MDX‐650, with automatic tool changer for a total cost of US$36,000.00. Figure 1(a) shows our MDX‐650 with a block of material in the jaws of its rotary unit allowing the block to rotate around its longest axis. In Fig. 1(b), the machine is cutting an object from the block of material. The constraints related to the machine capabilities needed to be addressed and compensated for by more advanced computer software that would take into account the limitations of the 4‐axis machine.

In this study we wanted to demonstrate the ability of SRP to perform as well as RP technology for the purpose of fabricating custom positioning fixtures in terms of accuracy and built capabilities. For this we used a modified Brown‐Roberts‐Wells (BRW) phantom base^(25)^ to measure the accuracy achievable in the build and assembly of the final fixture.

## II. MATERIALS AND METHODS

In order to assess the accuracy of our SRP machine for the fabrication of custom biopsy‐guides, we designed a series of nine guides that include a face mask, a biopsy needle holder, and connecting parts that link the needle holder to the face mask. A three‐point localizer (to be described later) was also added to the face mask to provide a reference to the BRW phantom base. The nine guides have identical face masks (same patient and same positioning surface). Each guide was designed to reach different targets that cover the full volume of a human head. The design process was as follows: 1) the three‐dimensional model of the patient's head was built from a specific series of diagnostic CT scans; 2) the contact surface of the patient's face with the face mask was selected using the graphic user interface (GUI) of our program and the selection was performed by painting the surface of the model; 3) a surgical plane was selected by choosing a target point, an entry point, as well as a length for the biopsy needle that should allow for space between the skin surface and the needle holder; 4) the specifications for the whole fixture were then generated by our software program.

The supporting computer code was developed in our laboratory. For code development we used the Insight Toolkit (ITK 1.8, Kitware Inc., Clifton Park, N Y, 2004). This is an image‐processing toolkit designed mainly for image segmentation and image registration. We also utilized the Visualization Toolkit (VTK 4.2, Kitware Inc., Clifton Park, NY, 2003). This toolkit is dedicated to 3D rendering and can work directly on image datasets or on 3D models such as polygonal surfaces. The user interface was developed using Qt (Qt 3.3.3, Trolltech Inc, Oslo, Norway, 2004).

### A. designing the biopsy guides

As mentioned previously, compared to a 3D printing process, the capabilities of our SRP machine limit the complexity of the objects that can be fabricated. Large objects will produce a longer built time and only one axis of rotation is allowed to change the orientation of the block of material. This latter constraint limits the cutting tool axis orientation relative to the object. The tool axis can be thought of as being constrained into a single plane that can be translated along the three main axes but can not change its orientation. Because two vectors always define a plane in space, we decided to break down the whole final fixture into as many parts as needed, as long as each part required only two cutting axes. With this in mind, it is always possible to place each part in an orientation that brings both cutting axes in the plan perpendicular to the rotation axis of our 4‐axis machine. The 4‐axis constraint was therefore eliminated by geometrical solutions implemented at the software level. The parts were designed with tightly fitting rectangular connectors – male or female – that can be manually snapped into each other and that allow for the assembling of the final fixture. Each male‐female set was standardized to make it unique within the build, allowing only one possible assembly of the whole fixture. Screws were used to prevent the assembled joints from disconnecting.

**Figure 2:**
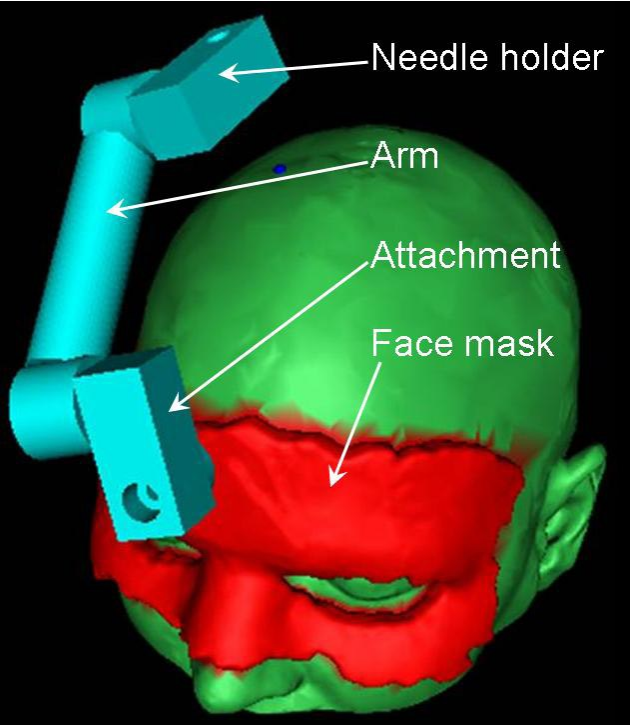
Computer model that represents a patient and a biopsy guide. The blue dot on the top of the patient's head represents the entry point. The face mask (in red) is positioned on the patient's face. The three other parts of the fixture (in cyan) are connected together to provide the correct orientation of the biopsy needle when introduced into the cylindrical hole of the needle holder.

In order to improve the time of build, we designed the whole fixture so that as many parts as possible have a standard (not requiring patient customization) geometry and can be fabricated in advance. In the case of a biopsy guide, four parts were required but only two needed to be customized to a specific patient. Figure 2 shows the computer‐generated model of a biopsy guide placed on the surface of the patient's skin. The face mask (customized) is shown in red. It is connected to the needle holder (standard) by the attachment (standard) and the arm (customized). The arm length and extremities were customized to provide the correct orientation and position of the needle holder. All parts were designed with male or female rectangular connectors that allow for the assembling of the final fixture. Note that the two standard parts can be selected by our software program from a series of several lengths in order to accommodate the central arm. This arm should be small enough to be built in a reasonable amount of time and should be positioned so that it does not touch the patient's skin. The two custom parts (the face mask and the arm) were then stored to disk as a stereolithography (STL) file format – a specific file format used by most rapid prototyping machines to represent the surface of an object – and transferred to the fabrication station.

### B. Building the biopsy guides

We selected the type of material according to the following properties: rigidity of the final guide, softness of the material to improve the cutting speed, and resistance to high temperature. While it is difficult to combine these three properties, there exist a few composite materials that provide a good compromise. Epoxical 650 and Necuron 700 are both distributed by Resaline, Inc. (Starling Heights, MI). They satisfy the rigidity criterion and they resist to a temperature of about 170 °C. Their hardness, though, increases the fabrication time. BUTTER‐BOARD R1/BB is distributed by Goldenwest Manufacturing Inc. (Cedar Ridge, CA). Its rigidity and softness allow a faster fabrication (i.e. easier cutting), but its heat defection temperature is only 120 °C. Though BUTTER‐BOARD cannot be used when sterilization is required, it is a great candidate for nonsterile parts and we decided to use it for the current study as all tests are done before the sterile field is prepared around the patient. Necuron 700 was also investigated and we found no noticeable difference in term of accuracy of the final guide.

The first step of the fabrication process was to analyze the shape of the object represented by the STL file and to calculate a tool path that would cut the parts from the block of material. This step was done using a software program provided with the milling machine: Mayka Pro (Mayka Pro 7.0, Picasoft, Theillay, France, 2006). Mayka Pro includes a graphic user interface that allows setting various parameters specific to the fabrication process. Among these parameters are: 1) the size of the block of material from which the parts will be cut, 2) the position of the parts within the block of material – the parts should not overlap and they should allow enough space between them so that the block remains rigid after the material is removed around the parts, 3) the rotation angle around the rotary unit axis that brings the cutting axis in alignment with the tool axis, 4) the cutting tool diameter, length, rotation per minute, and linear speed. Several tool sizes and end shapes were chosen, depending on the type of cut. For example, the contact surface of the face mask is better finished with a spherical tool end, whereas the different connectors will be more precise if they are finished with a cylindrical tool end. Furthermore, roughing cuts can be done with larger tools than finishing cuts, in order to reduce the built time. To avoid having to manually change the tools, our machine was equipped with an automatic tool changer that can select between four different tools.

**Figure 3:**
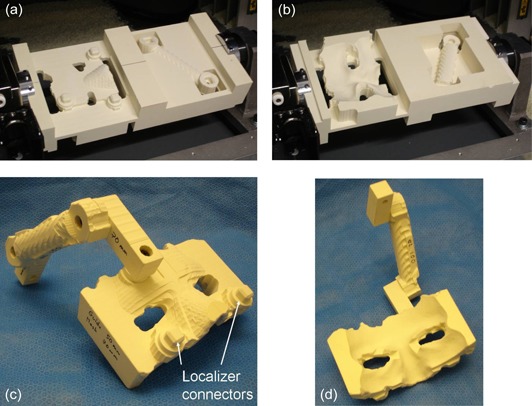
The biopsy guide fabricated by the MDX‐650: top side (a) of the block at the end of cutting; bottom side (b) of the block at the end of cutting. The assembled guide ‐ front side (c) showing the two squared connector that connect the fixture to the face mask localizer; back side (d) showing the surface of the face mask that position the fixture on the patient's face.

After all parameters were set using the user interface, Mayka Pro was asked to calculate the tool path and to generate a sequence of instructions compatible with the SRP machine interface protocol. The sequence of instructions was then sent to the machine using a standard parallel interface. At the end of fabrication, the block of material looks as shown in Fig. 3(a) and 3(b). In order to keep the rigidity of the block, the parts need to remain attached together until the end of the build. They were separated from the block using a handsaw and assembled with the standard parts fabricated in advance. Figures 3(c) and 3(d) show the whole fixture after assembly and ready for sterilization.

### C. Measuring the position of the biopsy needle

**Figure 4:**
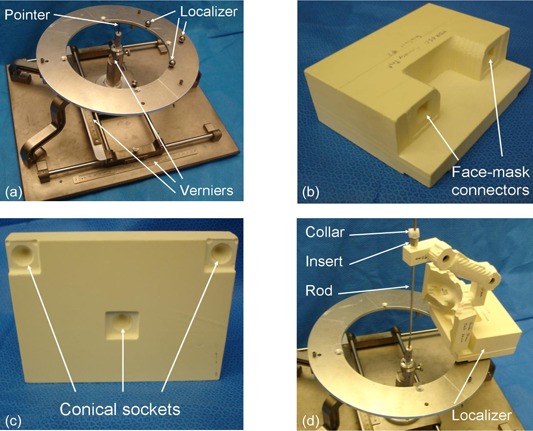
The measurement apparatus: the BRW phantom base (a) with its central pointer and the three‐sphere localizer used to receive the biopsy guide; the mask localizer (b) and (c) that will be attached to the biopsy guide – the three conical sockets seat on the three spheres during measurement; the biopsy guide (d) with the metallic rod introduced into the aluminum insert is attached to the localizer using the two connectors and placed on the BRW phantom base for measurement of the position of the rod tip.

To test the accuracy of the build, we used a BRW phantom base that we modified by incorporating a three‐sphere localizer (see Fig. 4(a)). The three spheres have a known geometry in the phantom base reference system and were used to position the face mask using the mask localizer shown in Fig. 4(b) and 4(c). The mask localizer is a standard part and has three conical sockets on its bottom side (Fig. 4(c)). The geometry of the three sockets matches the geometry of the three spheres on the phantom base. This allows for a unique position. Two rectangular female connectors were added to the mask localizer (Fig. 4(b)) in order to receive two male connectors that were incorporated to the front side of the face mask (Fig. 3(c)). At the center of the phantom base, a pointer can move along the three axes (Fig. 4(a)). The biopsy guide and the mask localizer were assembled and placed on the three spheres of the phantom base as shown in Fig. 4(d).

A rigid metallic rod with a sharp tip, simulating a biopsy needle, was placed into the needle holder. Its penetration depth into the holder was set to the planned biopsy length using an adjustable collar (Fig. 4(d)). Note that a metal insert of known geometry was introduced between the rod and the needle holder. Since different surgical tools may be utilized during the same surgery, the insert is used to adjust the diameter of the tool to the diameter of the holder. The tip of the pointer of the BRW phantom base was then moved along the three orthogonal directions – lateral (Lat), anterior‐posterior (A‐P), and axial (Ax) – until it touches the tip of the metallic rod. The three coordinates are then recorded using the vernier devices installed on the frame of the phantom base and compared with the coordinates of the target point. The three coordinate errors were calculated, as well as the Euclidean error (Euclidean distance between the tip of the rod and the target point).

The measurement was reproduced by five different experimenters and with nine different biopsy guides, each having a different target point. The nine targets were chosen to cover the whole space within a human head: one at the center of a $100\times 100\times 100\;{\text{mm}}^{3}$ cube, one at the center of each of the six faces of the same cube, and one at each of the two ends of the main diagonal of the cube. The second column of Table 1 shows the length of the biopsy needle (i.e. metallic rod). The length was given to the experimenters so that they could set the position of the white collar along the metallic rod (Fig. 4(d)).

**Table 1:** Summary of results for the Euclidean error from Calculation B.

*Guide*	*Rod length*	*Mean*	*Standard Deviation*	*Standard Deviation of the Mean (SDM)*	*Mean / SDM*
1	146	0.69	0.26	0.12	5.8
2	146	0.95	0.32	0.14	6.8
3	146	0.64	0.32	0.14	4.6
4	146	1.21	0.39	0.17	7.1
5	146	0.71	0.24	0.11	6.5
6	196	0.65	0.16	0.07	9.3
7	96	0.83	0.31	0.14	5.9
8	196	0.48	0.17	0.07	6.9
9	96	0.74	0.26	0.12	6.2
Average		0.77	0.21	0.07	11.0

Notes: The second column corresponds to the length of the biopsy needle (metallic rod for our test) as it must be set by the experimenters before they introduce the rod inside the aluminum insert. The mean, standard deviation, and standard deviation of the mean are calculated on the Euclidean error: 1) for each guide over the 5 experimenters (rows 2–10), and 2) over the 9 guides (last row). The last column is the ratio between the mean and the standard deviation of the mean. All dimensions, but the last column, are in mm.

### D. Statistical analysis

Sources for the guide error can be grouped into four Categories. Category 1: The precision of the different parts of the guide depends on the precision of the SRP machine and on the elasticity of the material during cutting. This source of error can be considered isotropic and affects the precision of the guides but not their accuracy (no bias). Category 2: Because of the tight fitting of the connectors, the deformation of the material during assemblage is also an important source of error. This error is expected to be mostly precision error, although, as the guides are very similar in shape, the deformation can be anisotropic and introduce some bias. Category 3: The phantom base and the mask localizer are part of the experimental apparatus and have their own accuracy that introduces a bias to the measurements. Category 4: Each measurement also introduces its own experimental error: connecting the guide to the mask localizer introduces deformation of the guide because of the tight fitting of the connectors; the metallic rod slides freely in its guide and can wobble around the guide axis; the metallic rod may not be perfectly straight and can wobble even more around the guide axis; adjusting the length of the metallic rod is subject to human error; the alignment of the tip of the pointer with the metallic rod tip is also subject to human error, as well as reading the three vernier devices. These sources of errors are clearly isotropic and should not introduce any bias. Note that the image acquisition and the extraction of the patient's skin surface by our software program do not affect the error in this experiment as only purely geometric surfaces are used to position the guide relative to the phantom base. Biases should be represented by a vector in space. As a consequence, the Euclidean error cannot be used to determine biases.

Errors of the type noted in Categories 1 and 2 above are due to the fabrication process and are the only ones that we would like to estimate in this study. Category 4 errors are purely experimental and we attempted to eliminate their extent by calculating the mean, standard deviation, and standard deviation of the mean of the three coordinate errors, for each of the nine guides and over the five experimenters. (We shall refer to this first calculation as Calculation A in the remainder of this text.) The mean is an estimator of the true coordinate error. As each coordinate error can clearly be considered normally distributed around its true value, 95% confidence and p‐values were calculated using the two tails of a Student distribution with 4 degrees of freedom. The 95% confidence interval tells us if the mean is a good estimator of the true error. The p‐value represents the probability of observing a coordinate error as extreme as the mean if the fabricated guide and the measurement apparatus were perfect (null hypothesis). It tells us whether we can be confident that the mean coordinate error indicates the presence of an actual error or if it can simply be a consequence of our small sample size. Next, the Euclidean errors were computed for each guide using the mean coordinate errors and then averaged over the nine guides to obtain an estimate of the overall fabrication and experimental error. Unfortunately, we will see later that our sample size is too small to trust the Euclidean error of each individual guide. As a consequence, we will not attempt to give a statistical interpretation of this Euclidean error.

Instead, we computed the Euclidean error for each of the 45 measurements and then calculated the mean, standard deviation, and standard deviation of the mean for each guide over the five experimenters. Next, the nine means were averaged to find an overall Euclidean error. (We shall refer to this as Calculation B in the remainder of this text.) Whether or not this calculation eliminates Category 4 errors is unclear to us because of the calculation of the norm of a vector before averaging the data. However, this calculation is expected to produce larger statistical fluctuation and can be seen as more conservative when interpreting the result than Calculation A. The Euclidian error is clearly bounded at zero and cannot be considered normally distributed. Therefore, and because of the small sample size of each of the two steps, we cannot calculate a 95% confidence interval or a p‐value. However, we can compare the means with their standard deviations for a qualitative estimate of our confidence in the results.

Category 3 errors are pure bias and, as they are related to the experimental apparatus, they should be eliminated from the errors attributed to the fabrication of the guide. Without bias, each coordinate error is assumed to be distributed around zero. Therefore, a non‐zero mean should be considered as bias and subtracted from each measurement before performing Calculation A or B. Note that Category 2 errors also introduce bias that cannot be isolated from Category 3 and should not be eliminated from our final calculation. However, an estimation of the effect of the bias on the overall Euclidean error will help us determine if we need to eliminate Category 3 bias in order to demonstrate the accuracy of our guides. To estimate the amount of bias in our experiment, we calculated the mean, standard deviation, and standard deviation of the mean for each of the coordinate errors over the 45 measurements. (We shall refer to this as Calculation C in the remainder of this text.) A 95% confidence interval and a p‐value were calculated using the two tails of the normal distribution (because of our large sample). The p‐value tells us if the mean coordinate error can be attributed to bias or is merely due to chance. We then subtracted the coordinate error means of Calculation C from the initial measurements and redid Calculation B with these unbiased values for comparison with the biased results.

## III. RESULTS & DISCUSSION

The individual means, confidence interval, and p‐values of Calculation A having little interest on their own, they are not reported here and our discussion will be general. Ten means out of the 27 (nine guides and three coordinates per guide) have a 95% confidence interval larger than 1 mm – one is actually larger than 2 mm. Eighteen means have their 95% confidence interval that includes the value zero (which would indicate no error). Only 9 out of the 27 means have a p‐value smaller than 5.0%. None of the nine guides has its three coordinate errors with a p‐value less than 5.0%. As a consequence, we conclude that our sample size (only five experimenters) is not large enough to assign any guide with a reliable positioning error. However, as mentioned previously, we did calculated the average Euclidean error over the nine guides and found a mean of 0.54 mm, a standard deviation of 0.22 mm and a standard deviation of the mean of 0.07 mm. Though these values cannot be statistically validated, they suggest a good accuracy of the fabrication process.

The results of Calculation B are presented in Table 1. Only one guide has a mean Euclidean error of more than 1 mm. For each guide, the ratio between the mean and its standard deviation is reported in the last column of Table 1. Though we were not able to calculate a p‐value for this calculation, the ratio indicates that the mean Euclidean error of each guide is too far from zero (at least 4.6 times the standard deviation of the mean) for its value to be attributed to chance. Therefore, each guide has a Euclidean error that our experiment can detect. The narrowness of the standard deviation of the mean also indicates that the mean can be seen as a good estimate of the true Euclidean error. In Calculation A, the sample size was declared too small to be able to give any confidence to each coordinate error. It may be dubious at first that in the case of the Euclidean error, the sample size is satisfactory whereas it was not for individual coordinate errors. This is a consequence of the calculation of the Euclidean error. The deviation of the coordinate error can spread on both sides of zero. When the distance from the true target point is computed from the three coordinates, the symmetrical spread is removed and the three coordinate errors are averaged. This reduces the amount of deviation.

The overall mean for the Euclidean error is 0.77 mm with a standard deviation of 0.21 mm and a standard deviation of the mean of 0.07 mm. This can be compared to the mean of Calculation A that was found to be 0.54 mm. The difference seems large (43%). We are not sure why these two values are not closer to each other. We believe that the lack of confidence in Calculation A is the reason, though there may be other reasons related to the calculation of the Euclidean distance. As we do not have enough confidence in Calculation A, we used the results of Calculation B for our best estimator of the average Euclidean error of our guides. Though our technical accuracy does not equal the 0.3 mm of most current stereotactic frame arc systems,^(^ ^26^ ^,^ ^27^ ^)^ it is on the same order (slightly larger) than the 0.5–0.6 mm for the frameless systems.^(^ ^27^ ^,^ ^28^ ^)^ As for a comparison with RP technology, a similar study conducted with our 3DP machine gave a mean Euclidean error of 1.92 mm with a standard deviation of 0.40 mm and a standard deviation of the mean of 0.12 mm. It is clear that the SRP technology, even though it requires an assemblage of five parts (four parts for the guide plus the mask localizer), provides a much greater accuracy than the 3DP technology. Other RP systems would probably provide an accuracy equivalent to SRP but at a much higher cost.

As an attempt to try to eliminate the bias due to the experimental apparatus (Category 3 error), Calculation C averaged each coordinate error over the 45 measurements. The results are presented in Table 2. The p‐values of column five indicate that only the mean along the lateral axis can be interpreted as a significant proof for bias in the experiment. However, assuming that the three coordinate axes are biased by the amount estimated by the means of Table 2, Calculation B was redone after subtracting the “assumed” bias from the experimental measure. The results are to be compared with the last row of Table 1. We found a mean of 0.73 mm, a standard deviation of 0.18 mm, and a standard deviation of the mean of 0.06 mm for these unbiased measurements. They are very similar to the biased results of Table 1, which shows that removing unwanted biases would not significantly change our results. This is an important finding to us because other non‐experimental biases exist in our measurement process (Category 2 errors) and removing only the unwanted biases would have been a difficult task. As a consequence, we consider the results of the last row of Table 1 to be a fair estimator for the average accuracy of our biopsy guides.

**Table 2:** Averaged measured error along each coordinate axis.

*Axis*	*Mean*	*Standard Deviation*	*Standard Deviation of the Mean (SDM)*	*P‐value (%)*
Lat	0.21	0.46	0.07	0.21
A‐P	−0.08	0.40	0.06	16.2
Ax	0.11	0.51	0.08	13.6

Notes: The average is performed over the 45 measurements. The p‐value represents the probability for the observed mean to be due to chance. All dimensions, except the p‐value, are in mm.

Another important parameter that we wanted to determine is the fabrication time for the lowest level of acceptable SRP unit. An estimation of each step was performed during the fabrication of the nine guides. Once the STL files are produced by the software program, it takes about 5 minutes to mount the block of material into the jaws of the machine. Setting the parameters for each tool path through the user interface of Mayka Pro requires a lot of attention. This task is repetitive and must be performed meticulously to avoid any mistake. Nineteen tool paths are needed for the two parts of a biopsy guide and 20 minutes is an average time for an experienced operator to perform this task. Then the computer calculates the 19 tool paths one at a time, which takes about 5 minutes, during which operator interventions are needed for each tool path. The actual fabrication takes approximately 75 minutes using BUTTER‐BOARD material. Finally, the parts must be separated from the block, fled, and assembled together using screws. This can be done in 10 minutes. Including image processing and treatment planning before fabrication as well as sterilization after fabrication, three‐and‐a‐half hours is a good estimation of the overall time for this level of SRP unit.

## IV. CONCLUSIONS

The series of tests proposed in this study demonstrated that programmable milling machines are suitable for building image‐guided biopsy guides. The fabricated guides can reproduce a preplanned target with submillimeter accuracy in most cases and with an estimated average error of 0.77±0.21mm. Though this error includes some experimental errors that should not be accounted for in the guide accuracy, our statistical evaluation of possible bias due to the experimental apparatus shows that, if this bias could be eliminated, our estimated accuracy would not improve significantly. Utilizing the most cost effective SRP unit on the market, the fabrication time allows for the guides to be ready within three‐and‐a‐half hours after diagnostic image acquisition. More robust SRP units are available. These units will decrease fabrication times as well as broaden the range of materials for mask and guide fabrication. The next level SRP units cost approximately twice the price of the unit used in these experiments. This next level unit would double the cutting accuracy with an anticipated overall increase in guide fabrication. In order to achieve a similar accuracy with other RP technologies such as Stereolithography or Inkjet printing, it is estimated that the cost would be on the order of US$100,000.00. The materials used in both techniques compared here have a very similar cost: about US$50.00 per guide. The main constraint of the SRP machine was the limitation due to the single axis of rotation allowed by the machine design. This constraint was eliminated by computer software intelligence that designs the whole fixture as a set of simple parts that are assembled and held together with tight adjusted connectors and screws. The assembling design also allows for reusing the same face mask with different tools that can be interchanged during surgery.

Some improvements can be made on the current technique toward faster build and more accurate guides. First, the standard parts used in this study were all built using our SRP machine. These parts, when their geometry is well defined, can be standardized and fabricated from a reusable material, such as stainless steel. Second, the parameters set in Mayka Pro through its user interface can be calculated by our computer software and sent directly to Mayka Pro through a computer interface. Such automatic interface has not been implemented in Mayka Pro yet, but future developments will make this interface possible and reduce both time and user mistakes during this phase of the process. A third improvement of the fabrication time can be achieved by prefabricating blocks of materials with a shape close to the final shape of the objects. Some composite materials offered by tooling manufacturers can be cast into a predefined shape. This would reduce the amount of material to be removed around the object and would considerably reduce the fabrication time. The two fabricated parts can also be built on two different machines and more robust machines can be used for a much faster build. With all these improvements, we estimated that the overall time could be reduced to one hour between image acquisition and surgery.

The experiments developed in this study demonstrate that SRP machines can fabricate accurate biopsy guides. However, to conclude the overall utility of the concept, the patient registration accuracy of the technique must be established. The previous study^(1)^ on RP machines only used a phantom to measure both technical and registration accuracy. The application of the face mask on the rigid face of a glass head provided for good positioning but it will be different when the rigid glass is replaced by the soft skin of a patient. The next step is to do clinical trials under a no‐risk IRB protocols. During these trials, the biopsy guides will be judged against FDA cleared state‐of‐the‐art image guidance systems. These trials will enroll patients scheduled to undergo stereotactic biopsy, ventriculoperitoneal (VP) shunts and for the insertion of deep brain stimulation electrodes.

## ACKNOWLEDGEMENTS

This work was supported in part by Grant #5R01EB2573‐3 from the National Institute of Health NIH‐NIBIB. We also thank Atchar Sudhytadhom, Heeteak Chung, and Barbara Garita for their participation in the accuracy test.
